# Defining the Intestinal eCBome and Oxylipin Signaling Systems in a TDP‐43 Mouse Model of Frontotemporal Dementia

**DOI:** 10.1096/fj.202502312RR

**Published:** 2026-01-12

**Authors:** Hayatte‐Dounia Mir, Giada Giorgini, Irene Santos‐García, Elizabeth Dumais, Carmen Rodríguez‐Cueto, Eva de Lago, Cristoforo Silvestri, Nicolas Flamand, Javier Fernández‐Ruiz, Vincenzo Di Marzo

**Affiliations:** ^1^ Département de Médicine (DMED), Centre de Recherche de l'Institut Universitaire de Pneumologie et Cardiologie (CRIUCPQ), Faculté de Médicine (FMED) Université Laval Québec Canada; ^2^ Canada Research Excellence Chair in the Microbiome‐Endocannabinoidome Axis in Metabolic Health (CERC‐MEND) Université Laval Québec Canada; ^3^ Unité Mixte Internationale en Recherche Chimique et Biomoléculaire sur le Microbiome et son Impact sur la Santé Métabolique et la Nutrition (UMI‐MicroMeNu) entre l'Université Laval Québec Canada; ^4^ Institute of Biomolecular Chemistry (ICB‐CNR) Consiglio Nazionale Delle Ricerche Pozzuoli Italy; ^5^ Departamento de Bioquímica y Biología Molecular, Instituto Universitario de Investigación en Neuroquímica Facultad de Medicina, Universidad Complutense Madrid Spain; ^6^ Centro de Investigación Biomédica en Red de Enfermedades Neurodegenerativas (CIBERNED) Madrid Spain; ^7^ Instituto Ramón y Cajal de Investigación Sanitaria (IRYCIS) Madrid Spain; ^8^ Institut sur la Nutrition et Les Aliments Fonctionnels (INAF) and NUTRISS Center Université Laval Québec Canada; ^9^ Faculté des Sciences de l'Agriculture et de l'Alimentation (FSAA) Université Laval Québec Canada; ^10^ Centre Nutrition, Santé et Société (NUTRISS) Université Laval Québec Canada

**Keywords:** endocannabinoidome, FAAH, frontotemporal dementia, gut‐brain axis, microbiota, short‐chain fatty acids, TDP‐43

## Abstract

Frontotemporal dementia (FTD) is a group of early onset and progressive disorders, characterized by degeneration in the frontal and temporal lobes, and subsequent deterioration in cognition, personality, social behavior, and language, with aggregates of the RNA‐binding protein TDP‐43 being present in ~45% of the cases. We reported alterations of the endocannabinoidome (eCBome) in the brain of a TDP‐43 mouse model of FTD. Here we investigated the small intestinal eCBome, oxylipins, and, preliminarily, the gut microbiome. The duodenum, jejunum, and ileum of TDP‐43 overexpressing versus wildtype mice were investigated. Lipid mediators were measured by HPLC‐MS/MS, and mRNA expression of genes involved in eCBome mediator action and metabolism, or intestinal permeability and inflammation, was analyzed by qPCR. Intestinal content microbiota composition and fecal short‐chain fatty acids were studied by 16S DNA sequencing and GC‐FID, respectively. Alterations were observed in TDP‐43 mice for polyunsaturated fatty acids, *N*‐acyl‐ethanolamines, and oxylipins in the duodenum and the jejunum, and for oxylipins and 2‐monoacylglycerols in the ileum. Regarding the receptors, mRNA expression of *Cnr1* and *Gpr119* was increased in the ileum, and that of *Pparg* in the duodenum, where *Gpr55* was instead down‐regulated. Regarding the enzymes, *Faah* and *Napepld* expression was increased in the ileum. Preliminary gut microbiota data suggest increases of *Paraprevotella* and *Monoglobus* in the feces, of *DNF00809* in the ileum, and of *Butyricicoccus*, *Candidatus_Arthromitus*, and *Oscillospira* in the cecum, where *Paraprevotella*, *Mucispirillum*, and *Akkermansia* were instead decreased. Fecal acetic, butyric, and isobutyric acid were reduced. We suggest the existence of lipid signal‐mediated gut‐brain interactions in FTD.

## Graphical abstract

TDP‐43 overexpressing mice, a genetic model of frontotemporal dementia, exhibit dramatic changes in their small intestinal endocannabinoidome members, in terms of both altered concentrations of lipid mediators and expression of some of their receptors (*Cnr1, Gpr119, Gpr55, Pparg*) and metabolic enzymes (*Napepld, Faah*). Also, the levels of some oxylipins were altered. These lipid mediator changes might contribute to the phenotype of TDP‐43 mice.

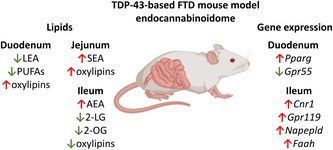

**Abbreviations**
10,17‐DiHDHA (PDX): 10,17‐dihydroxy‐docosahexaenoic acid (Protectin DX)
11‐HETE: 11‐hydroxy‐eicosatetraenoic acid
12‐HEPE: 12‐hydroxy‐eicosapentaenoic acid
12‐HETE: 12‐hydroxy‐eicosatetraenoic acid
12‐HETrE: 12‐hydroxy‐eicosatrienoic acid
12‐KETE: 12‐oxo‐eicosatetraenoic acid
13‐HODE: 13‐hydroxy‐octadecadienoic acid
13‐HODE‐EA: 13‐hydroxy‐octadecadienoic acid ethanolamide
13‐HODE‐G: 13‐hydroxy‐octadecadienoic acid glycerol
13‐HOTrE: 13‐hydroxy‐octadecatrienoic acid
13‐KODE: 13‐oxo‐octadecadienoic acid
14‐HDHA: 14‐hydroxy‐docosahexaenoic acid
15‐HEPE: 15‐hydroxy‐eicosapentaenoic acid
15‐HETE: 15‐hydroxy‐eicosatetraenoic acid
15‐HETrE: 15‐hydroxy‐eicosatrienoic acid
17‐HDHA: 17‐hydroxy‐docosahexaenoic acid
17‐HDPA: 17‐hydroxy‐docosapentaenoic acid
17‐oxo‐DHA: 17‐oxo‐docosahexaenoic acid
18‐HEPE: 18‐hydroxy‐eicosapentaenoic acid
1a,1b‐dihomo PGF2α: 1a,1b‐dihomo prostaglandin F2α
2‐AG: 2‐arachidonoyl‐glycerol
2‐DHG: 2‐docosahexaenoyl‐glycerol
2‐DPG: 2‐docosapentaenoyl‐glycerol
2‐LG: 2‐linoleoyl‐glycerol
2‐MAGs: 2‐monoacyl‐glycerols
2‐OG: 2‐oleoyl‐glycerol
4‐HDHA: 4‐hydroxy‐docosahexaenoic acid
5,12‐DiHETE: 5,12‐dihydroxy‐eicosatetraenoic acid
5‐HETE: 15‐hydroxy‐eicosatetraenoic acid
6‐keto PGF1a: 6‐keto prostaglandin F1α
7‐HDHA: 7‐hydroxy‐docosahexaenoic acid
8‐HETE: 8‐hydroxy‐eicosatetraenoic acid
9‐HODE: 9‐hydroxy‐octadecadienoic acid
AA: arachidonic acid
AD: Alzheimer's disease
AEA: endocannabinoid anandamide
ALS: amyotrophic lateral sclerosis
ASD: autism spectrum disorder
ASVs: amplicon sequence variants
BDNF: brain‐derived neurotrophic factor
bvFTD: behavioral variant FTD
CB2: cannabinoid type‐2
CNS: central nervous system
DGLA: dihomo‐γ‐Linolenic Acid
DHA: docosahexaenoic acid
DHEA: N‐docosahexaenoyl‐ethanolamine
DPA (n‐3): docosapentaenoic acid (omega‐3)
DPA (n‐6): docosapentaenoic acid (omega‐6)
DPEA (n‐3): N‐docosapentaenoyl‐ethanolamine (omega‐3)
DPEA: N‐docosapentaenoyl‐ethanolamine
eCB: endocannabinoid
eCBome: endocannabinoidome
EOD: early‐onset dementia
EPA: eicosapentaenoic acid
FAAH: fatty acid amide hydrolase
FTD: frontotemporal dementia
GC‐FID: gas chromatography‐flame ionization detection
GPR119: G protein‐coupled receptor 119
GPR18: G protein‐coupled receptor 18
GPR55: G protein‐coupled receptor 55
HPLC‐MS/MS: high‐performance liquid chromatography‐mass spectrometry/mass spectrometry
IL‐17: interleukine‐17
IL‐6: interleukine‐6
ISDT: internal standard
LA: linoleic acid
LEA: *N*‐linoleoyl‐ethanolamine
LTB4: leukotriene B4
MIND: mediterranean‐DASH intervention for neurodegenerative delay
NAEs: *N*‐acyl‐ethanolamines
NAPE‐PLD: *N*‐acyl‐phosphatidyl‐ethanolamine‐selective phospholipase D
OA: oleic acid
PCoA: principal coordinate analysis
PEA: *N*‐palmitoyl‐ethanolamine
PGE1: prostaglandin E1
PGE2: prostaglandin E2
PGF2a: prostaglandin F2α
PPARα: peroxisome proliferator‐activated receptor alpha
PUFAs: polyunsaturated fatty acids
RVD5: resolvin D5
RVE4: resolvin E4
SAMP8: senescence‐accelerated mouse‐prone 8
SCFAs: short chain fatty acids
SD: standard deviation
SEA: *N*‐stearoyl‐ethanolamine
SPMs: specialized pro‐resolving mediators
TRPV1: transient receptor potential cation channel subfamily V member 1
WT: wild‐type

## 1 Introduction

With 4.1 cases per 100 000 subjects per year, frontotemporal dementia (FTD) is one of the most common causes of early dementia in the world [1]. Different forms of FTD exist, such as early and progressive ones, which may be associated with differences in clinical signs, also in relation to the etiology and, in particular, the protein (e.g., Tau, TDP‐43) whose alteration elicits the disease. These forms are characterized by atrophy of the frontal and temporal lobes. These atrophies are thought to be caused by synaptic and neuronal loss, reactive gliosis, and microvacuolization [2]. This specific degeneration of the frontal and temporal lobes is responsible for several clinical signs including impairment of executive functions such as working, episodic and semantic memory, cognitive flexibility, attention, and emotional processes, as well as cognitive functions, such as planning, decision‐making, social cognition, and language processing [2, 3].

At the histopathological level, FTD is characterized by the aggregation of toxic proteins, which are immunologically detectable and generated by alterations in protein homeostasis, in both glial cells and neurons [4]. These cytoplasmic inclusions allow for the classification of several forms of FTD due to: (i) TDP‐43 (40%–45%), (ii) Tau (40%–45% of genetic cases), (iii) FUS (5%–10%), and (iv) UPS (1%) [2, 5, 6]. Its main histopathological hallmark is the presence of cytoplasmic inclusions as a result of the delocalization of the nuclear and functional ribonucleoprotein TDP‐43. This protein is known to regulate the transcription, stability, transport, and processing of RNA [7]. In FTD, these TDP‐43 protein inclusions can be hyperphosphorylated, ubiquitinated, and/or partially cleaved and are located mainly in the prefrontal cortex, the dentate gyrus of the hippocampus, and the striatum, leading to intense atrophy of the frontal and temporal lobes accompanied by gliosis and hippocampal sclerosis [7].

Unfortunately, there is currently no approved neuroprotective treatment for FTD. An interesting neuroprotective approach may be the use of endocannabinoid (eCB) signaling modulators. This regulatory system is largely present in the central nervous system (CNS), where it orchestrates key functions in the control of homeostasis, integrity, and survival of neurons and other cells [8]. eCBs are being investigated in different neurodegenerative and neuroinflammatory disorders (e.g., Parkinson's disease, Alzheimer's disease, Huntington's chorea, multiple sclerosis, amyotrophic lateral sclerosis), although mostly at the preclinical stage with only a few clinical trials already finalized or currently in progress [8]. Recent experimental evidence has related Tau‐dependent FTD with dysregulation in eCB signaling [9], which may support the pharmacological modulation of certain proteins of this system (e.g., cannabinoid type‐2 [CB2] receptors) as a promising disease‐modifying therapy in this form of FTD [9, 10, 11]. Such potential has been previously investigated by us in a mouse model of TDP‐43‐dependent FTD [12], with promising results. Indeed, we showed that the inhibition of fatty acid amide hydrolase (FAAH), a catabolic enzyme for the endocannabinoid anandamide (AEA) and its congeners, the *N*‐acyl‐ethanolamines (NAEs), which are part of the extended eCB system, or endocannabinoidome (eCBome) [13], improved the cognitive deficit and reduced neuronal loss and inflammatory events in this preclinical model [12]. These results have more recently been completed with a new study dealing with the involvement of CB1 and/or CB2 receptors in these effects [14]. At the same time, we observed that these TDP‐43‐overexpressing mice exhibited a decrease of FAAH in the prefrontal cortex and the hippocampus, an increase in the NAE biosynthetic enzyme *N*‐acyl‐phosphatidyl‐ethanolamine‐selective phospholipase D (NAPE‐PLD) in the hippocampus, but only modest elevations in brain NAE (including AEA) levels. Importantly, the role of the intestinal eCBome and the gut‐brain axis, and of intestinal lipid signaling homeostasis in general, has not been investigated in this model of FTD, and is also poorly known for other forms of this disease [13].

Another “provider” of host‐influencing chemical signals that is being increasingly recognized to play a role in brain disorders is the gut microbiome [15]. In fact, the plethora of microorganisms inhabiting the gut produce small metabolites, such as the short chain fatty acids (SCFAs), that produce strong effects on the gut‐brain axis [16], as well as on the eCB system and the eCBome [17, 18]. In this context, we previously showed that the intestinal and brain eCBome of germ‐free mice is altered compared to conventionally raised mice [19, 20], suggesting a potential role of the gut microbiota‐eCBome axis in brain dysfunctions such as those observed in FTD.

For all these reasons, we have undertaken here a study aimed at investigating if the intestinal eCBome and microbiome are also altered in TDP‐43‐overexpressing mice, and if so, if they are changed in a manner that may be related to the development of the pathology. Since we have developed a targeted lipidomics analytical method that allows for the quantification not only of tens of eCBome mediators, such as NAEs, the other endocannabinoid 2‐arachidonoyl‐glycerol (2‐AG) and its congeners, the 2‐monoacyl‐glycerols (2‐MAGs), but also several of their corresponding polyunsaturated fatty acids (PUFAs) and PUFA, NAE, and 2‐MAG oxidation products, we have investigated the potential alterations in these lipid mediators in the small intestine of TDP‐43‐overexpressing mice. The results obtained shed some light on the potential implication of the eCBome and other lipid signaling systems, including those derived from gut microbiota, in the pathophysiology of FTD.

## 2 Materials & Methods

### 2.1 Animals

Animal experiments were conducted in a TDP‐43‐based FTD mouse model that overexpresses TDP‐43 protein exclusively in the forebrain under the control of the CaMKIIα promoter. In particular, the increases affect the medial prefrontal cortex and the hippocampus, with elevations close to 50% over wildtype mice levels [12, 21]. Mice (90 days old males) were housed in the animal facilities (CAI‐Animalario, Faculty of Medicine, Complutense University of Madrid, ref. ES280790000086) under controlled photoperiod (08:00–20:00 light) and temperature (22°C ± 1°C), and with free access to standard diet (LASQCdiet Rod18‐H from Altromin Spezialfutter Gmbh & COoKG, Lage, Germany) and water. All experiments were conducted according to local and European rules (directive 2010/63/EU), as well as conformed to ARRIVE guidelines. They were approved by the ethical committees of our university and the regulatory institution (ref. PROEX 056/19 and PROEX 201.8/22). Mice were housed together based on their genotype (*n* = 6 WT mice and *n* = 5 TDP‐43 mice per cage). WT mice were bred separately from the TDP‐43 mice, although in the same animal facility and period of time and with the same diet.

Fecal pellets were collected from each mouse of each genotype at the same time of the day and same day. At the end of the experiments, mice were killed by rapid decapitation and intestinal tissues dissected within 5 min, flushed with saline to collect microbiota‐containing contents and frozen at −80°C. Intestinal section contents were also immediately stored at −80°C.

### 2.2 Lipid Mediator Profiling by HPLC‐MS/MS

The analysis of 2‐MAGs, NAEs, PUFAs, 2‐MAG‐derived oxylipins, NAE‐derived oxylipins, octadecanoids, docosanoids, and eicosanoids such as prostaglandins, prostaglandin‐glycerols, prostamides, leukotrienes was performed by mixing ground tissues (~10 mg) with 500 μL of Tris–HCl (50 mM, pH 7). The full list of compounds is available in Figure S1. The samples were then vortexed and 500 μL of methanol solution was added to each sample. Methanol solution consisted of methanol (0.55 mL per sample), ISDT solution (10 μL per mL of methanol) and glacial acetic acid (11.5 μL per mL of methanol). For each sample, 1 mL of chloroform was then added and vortexed for 60 s at 3000 rpm, followed by centrifugation at 4000 rpm for 5 min at low brakes at room temperature. After the centrifugation, the chloroform phase containing the lipids was collected and 1 mL of chloroform was added again to each sample followed by a vortex and centrifugation step (same parameters as above). The chloroform fraction was then evaporated using a vacuum concentrator (SpeedVac, SPD130DLX). Once dry, the lipids were resuspended in a solution containing 30 μL of solvent A (H_2_O with 1 mM of ammonium acetate and 0.05% of acetic acid) and 30 μL of solvent B (MeCN:H_2_O (95:5) with 1 mM of ammonium acetate and 0.05% of acetic acid) and vortexed for 30 s before being transferred to a glass vial with insert and stored at −80°C until further analysis. Forty microliters of the extracted samples were injected onto an HPLC column (Kinetex C8, 150 × 2.1 mm, 2.6 μm, Phenomenex, Torrance, CA, USA) and eluted at a flow rate of 0.4 mL/min using a discontinuous gradient from 35% to 75% of solvent B in 10 min, from 75% to 95% in 10 s and held to 95% for 5 min. The HPLC system was interfaced with the electrospray source of a Shimadzu 8050 triple quadrupole mass spectrometer. Mass spectrometric analysis was performed in the negative or positive ion mode using multiple reaction monitoring for the specific mass transition of each lipid. The lipids were quantified using calibration curves that were extracted as described above. Of note, samples were analyzed in a blind manner and only peaks having the same retention time as the purified compounds of interest and showing a signal‐to‐noise ratio > 5 were considered for quantification. In the case of monoacylglycerols, the data are presented as 2‐MAGs but represent the combined signals from the *sn‐*2‐ and −1(3)‐isomers.

### 2.3 Short‐Chain Fatty Acid Profiling by GC‐FID

Fecal samples were collected and kept at −80°C until SCFA extraction and measurement by gas chromatography. The feces were dissolved in water and fecal suspensions were homogenized for 2 min with a Bead Ruptor 12 (Omni International, Kennesaw, GA, USA), then centrifuged at 18 000 *g* for 10 min at 4°C. The supernatant was collected and spiked with a solution containing an internal standard (4‐methylvaleric acid) and H_3_PO_4_ 10% to obtain a pH around 2.

A volume of methyl tert‐butyl ether equivalent to the volume of diluted sample was added to extract SCFAs by vortexing 2 min. Samples were then centrifuged at 18 000 *g* for 10 min at 4°C and the organic phase was transferred to a glass vial. SCFA analysis was performed on a GC‐FID system (Shimadzu), consisting of a GC 2010 Plus gas chromatograph equipped with an AOC‐20s autosampler, an AOC‐20i auto‐injector, and a flame ionization detector. The system was controlled by GC Solution software. One microliter of the organic phase was injected in split mode into a Nukol capillary GC column (30 m × 0.25 mm id, 0.25 μM film thickness, Supelco Analytical) and hydrogen was used as carrier gas. The injector and detector were set to 250°C. The oven temperature was initially programmed at 60°C, then increased to 200°C at 12°C/min, hold 2 min. SCFAs were quantified using a 5‐point calibration curve prepared with a mix of standards (acetic acid, propionic acid, butyric acid, isobutyric acid, valeric acid, and isovaleric acid) extracted following the same procedure as samples.

### 2.4 Quantification of mRNA Expression Levels by Real‐Time RT‐qPCR

For each mouse, the duodenum, jejunum, and ileum were stored in RNA later after dissection until the day of the RNA extraction. RNA was extracted using QIAzol lysis reagent (#79306, Qiagen, Hilden, Germany) and its manufacturer's protocol. Tissues were processed randomly and blindly. The concentration and purity of RNA were determined by measuring the absorbance at 260, 280 and 230 nm. One microgram of total RNA was reverse transcribed using a High‐Capacity cDNA Reverse Transcription Kit (#4368814, Applied Biosystems, CA, USA) in a reaction volume of 20 μL. Real‐time RT‐qPCR was used to quantify the mRNA of genes encoding (i) receptors and enzymes of the eCBome (*Cnr1*, *Cnr2*, *Ppara*, *Pparg*, *Trpv1*, *Gpr18*, *Gpr55*, *Gpr119*, *Faah*, *Napepld*, *Mgll*, *Alox5*, *Alox12*), (ii) permeability markers (*Tjp1*, *Dsg2*), and (iii) inflammation markers (*Tnfa*, *Il1b*) using BrightCycle Universal SYBR Green qPCR Mix (#RK21219, Abclonal, Woburn, MA, USA) and the CFX Opus 384 Real‐Time PCR system (#12011452, BioRad, Hercules, CA, USA). The primer sequences are available in Table S3. Data normalization was performed using the *Gapdh* housekeeping gene, and the relative quantification was assessed by the calculation of 2^−∆∆Ct^ using the Excel program.

### 2.5 Microbial Composition Assessment

DNA was isolated from fecal samples, as well as from the contents of the caecum, jejunum, and ileum, using the DNeasy 96 PowerSoil Pro QIAcube HT Kit (Qiagen, Hilden, Germany) following the manufacturer's protocol. DNA concentrations were quantified fluorometrically using the Quant‐iT PicoGreen dsDNA (Thermo Fisher Scientific, MA, USA). Possible contamination of sequencing results from DNA of intestinal cells or other sources was ruled out by the fact that sequencing libraries were prepared specifically targeting the V3–V4 region of the 16S rRNA gene using Illumina‐specific primers (forward: 5′‐TCGTCGGCAGCGTCAGATGTGTATAAGAGACAGCCTACGGGNGGCWGCAG‐3′; reverse: 5′‐GTCTCGTGGGCTCGGAGATGTGTATAAGAGACAGGACTACHVGGGTATCTAATCC‐3′) along with the Illumina DNA Prep (Sets A–D) kit (Illumina, USA). To control for bacterial DNA contamination of reagents, a parallel DNA extraction protocol was run without the addition of lumen content; no DNA was detected in any negative control. Though unlikely, as great care was taken during dissection using sterile tools, small intestinal samples may have been environmentally contaminated. However, the type of contaminations potentially affecting these samples are concerned with bacterial taxa that are not normally found in the mouse gut microbiome and/or would likely affect in the same manner both WT and FTD mice. After library normalization and pooling to 4 nM, denaturation and dilution to 8 pM were carried out. Paired‐end sequencing (2 × 300 bp) was conducted using the MiSeq Reagent Kit v3 (600‐cycle format) on the Illumina MiSeq platform. On‐instrument image analysis and base calling were performed automatically. The resulting sequences were processed using the DADA2 pipeline [22], following the recommended workflow. Amplicon sequence variants (ASVs) were taxonomically classified using the SILVA SSURef 138.1 NR database (March 2021) under default parameters [23], and identified eukaryotic sequences were eliminated. Downstream analyses were conducted via MicrobiomeAnalyst [24, 25]. Following preprocessing, 44 low‐variance features were excluded using the interquartile range method, resulting in a final dataset of 395 features. Microbial community composition was evaluated through alpha‐ and beta‐diversity metrics, with Bray–Curtis dissimilarity used for beta‐diversity. Principal coordinate analysis (PCoA) and pairwise PERMANOVA were then employed to assess group‐level differences. Tukey's multiple comparisons of means was also used for alpha‐diversity.

### 2.6 Statistical Analyses

For the lipidomics analyses in intestines as well as for fecal SCFAs analyses, the unpaired *t*‐test was used. For lipidomics, values equal to zero were replaced by 80% of the lowest concentration of the compound in the region of interest. *T*‐test was applied to log‐transformed data, and medians and interquartile ranges are represented in the graphs. In addition, to study gene expression in the small intestine, the Mann–Whitney test was used, and mean and SD are represented in the graphs. A global analysis of the microbiome was performed using the online platform MicrobiomeAnalyst‐Pro [25]. Data were either represented as median and interquartile range (for lipidomics), mean ± SD (for gene expressions and SCFA), and the level of significance was set at *p* < 0.05. Statistical analyses were performed with the GraphPad Prism software (version 10.4.0, La Jolla, CA, USA).

## 3 Results

### 3.1 FTD Mice Show Altered Levels of Endocannabinoid‐Like Mediators and Oxylipins in the Small Intestine Compared to WT Mice

**FIGURE 1:**
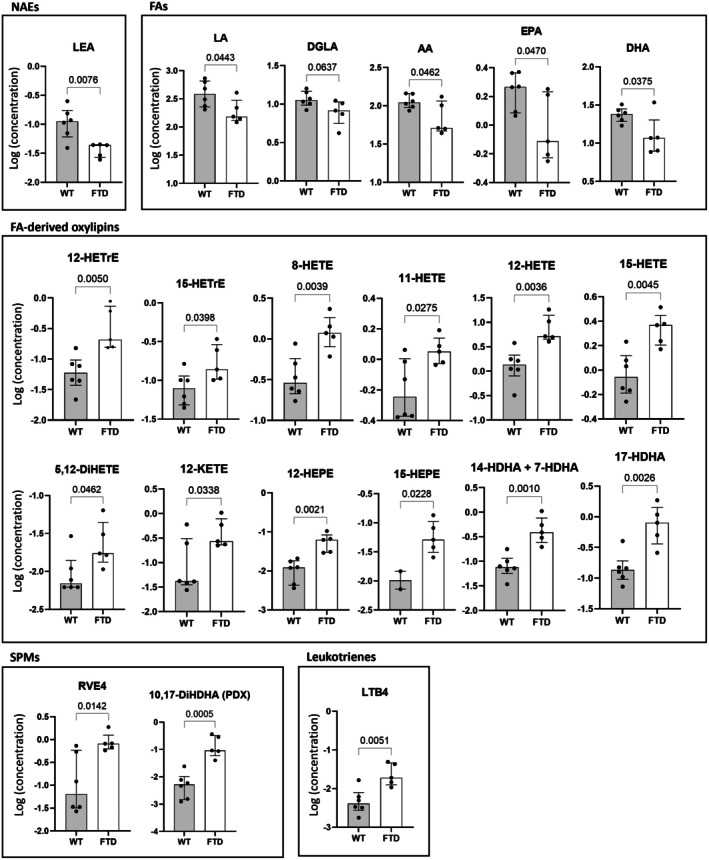
FTD mice show altered levels of endocannabinoid‐like mediators and oxylipins in the duodenum as compared to WT mice. Only those mediators for which the difference between the two groups was statistically significant (*p* < 0.05) or trending towards significant (*p* < 0.1) are shown.

In the duodenum (Figure 1) we observed a reduction in the levels of the NAE, *N*‐linoleoyl‐ethanolamine (LEA); this correlated with the concomitant reduction in its ultimate precursor linoleic acid (LA) and other PUFAs (arachidonic acid [AA], eicosapentaenoic acid [EPA], and docosahexaenoic acid [DHA]) in FTD mice. FTD mice also showed changes in the levels of numerous eicosanoids and docosanoids, with increased levels of 15‐HEPE, 12‐HEPE, 12‐HETE, 15‐HETE, 11‐HETE, 8‐HETE, 17‐HDHA, 14‐HDHA, and/or 7‐HDHA, 12‐KETE, 15‐HETrE, 12‐HETrE, 5,12‐DiHETE, RVE4, 10,17‐DiHDHA, and leukotriene B4 (LTB4).

**FIGURE 2:**
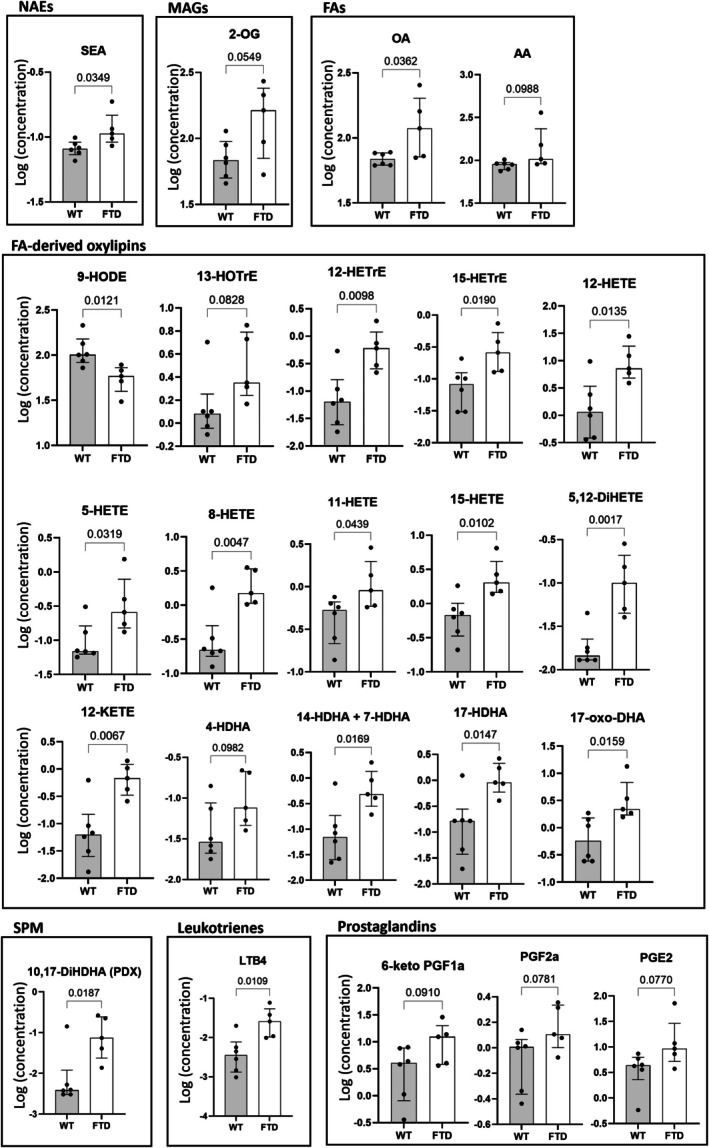
FTD mice show altered levels of endocannabinoid‐like mediators and oxylipins in the jejunum compared to WT mice. Only those mediators for which the difference between the two groups was statistically significant (*p* < 0.05) or trending towards significant (*p* < 0.1) are shown.

In the jejunum (Figure 2), we observed significant increased levels of the NAE, *N*‐stearoyl‐ethanolamine (SEA), and of oleic acid (OA) in FTD mice, while for 2‐OG and AA the increases showed only a statistical trend. The FTD mice also showed significant increases in the levels of several oxylipins, such as 12‐HETE, 15‐HETE, 11‐HETE, 8‐HETE, 17‐HDHA, 14‐HDHA, and/or 7‐HDHA, 12‐KETE, 5‐HETE, 17‐oxo‐DHA, 15‐HETrE, 12‐HETrE, 13‐HOTrE, and 5,12‐DiHETE, and a decrease of 9‐HODE. The oxylipin 4‐HDHA did not show a significant increase but only a trend. 10,17‐DiHDHA (PDX) and LTB4 were significantly increased, while the prostaglandins (6‐keto PGF1a, PGF2a, and PGE2) only showed a trend.

**FIGURE 3:**
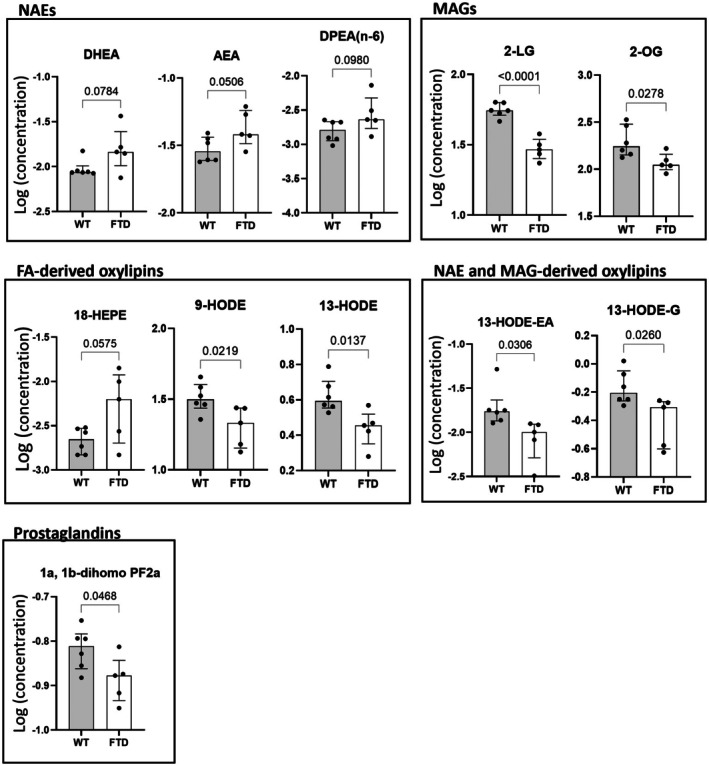
FTD mice show altered levels of endocannabinoid‐like mediators, oxylipins and prostaglandins in the ileum compared to WT mice. Only those mediators for which the difference between the two groups was statistically significant (*p* < 0.05) or trending towards significant (*p* < 0.1) are shown.

In the ileum (Figure 3), FTD mice showed increased levels of AEA (while for DHEA and DPEA the increases were a statistical trend) and reduced levels of two 2‐MAGs, 2‐LG and 2‐OG. These mice also showed reduced levels of PUFA‐derived oxylipins, such as 9‐HODE and 13‐HODE (and a non‐statistically significant trend for 18‐HEPE); of NAE‐ or 2‐MAG‐derived oxylipins, that is, 13‐HODE‐EA and 13‐HODE‐G; and 1a,1b‐dihomo PGF_2α_.

### 3.2 FTD Mice Show Altered eCBome Gene Expression in the Small Intestine Compared to WT Mice

**FIGURE 4:**
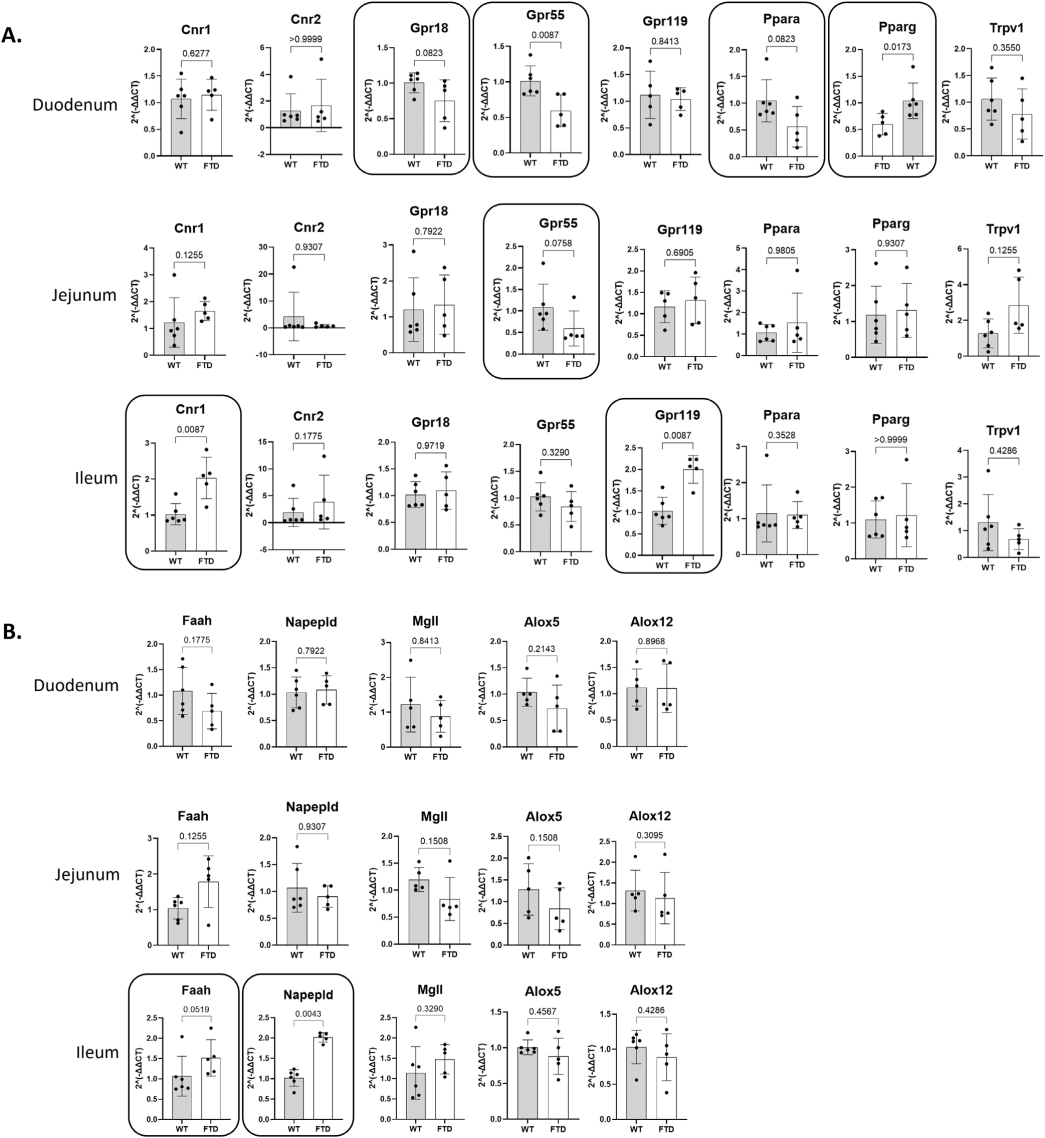
Expression of genes related to eCBome (A) receptors and (B) enzymes in the small intestine. Only those genes for which the difference between the two groups was statistically significant (*p* < 0.05) or trending towards significant (*p* < 0.1) are shown.

We studied the gene expression of receptors (*Cnr1*, *Cnr2*, *Gpr18*, *Gpr55*, *Gpr119*, *Ppara*, *Pparg*, *Trpv1*) and enzymes (*Faah*, *Napepld*, *Mgll*, *Alox5*, *Alox12*) related to the eCBome as well as markers of intestinal permeability (*Tjp1*, *Dsg2*, *Ocln*, *Cdh1*) and inflammation (*Tnfa*, *Il1b*) in the small intestine of FTD and WT mice (Figure 4A,B and Figure S1).

In the duodenum, FTD mice showed reduced expression of *Gpr55* and increased expression of *Pparg*. They also showed a statistical trend towards the reduction of *Gpr18, Ppara*, and *Dsg2* (*p* = 0.0887) expression.

In the jejunum, FTD mice did not show any significant changes but only a statistical trend towards a reduced expression of *Gpr55* and increased expression of *Ocln* (*p* = 0.0952).

In the ileum, FTD mice showed a significant increase of *Cnr1*, *Gpr119*, and *Napepld* and *Faah* expression. They also showed statistical trends for the increased expression of *Cdh1* (*p* = 0.0823).

### 3.3 Potentially Altered Fecal and Gut Microbiota Composition in FTD Mice Compared to WT Mice

**FIGURE 5:**
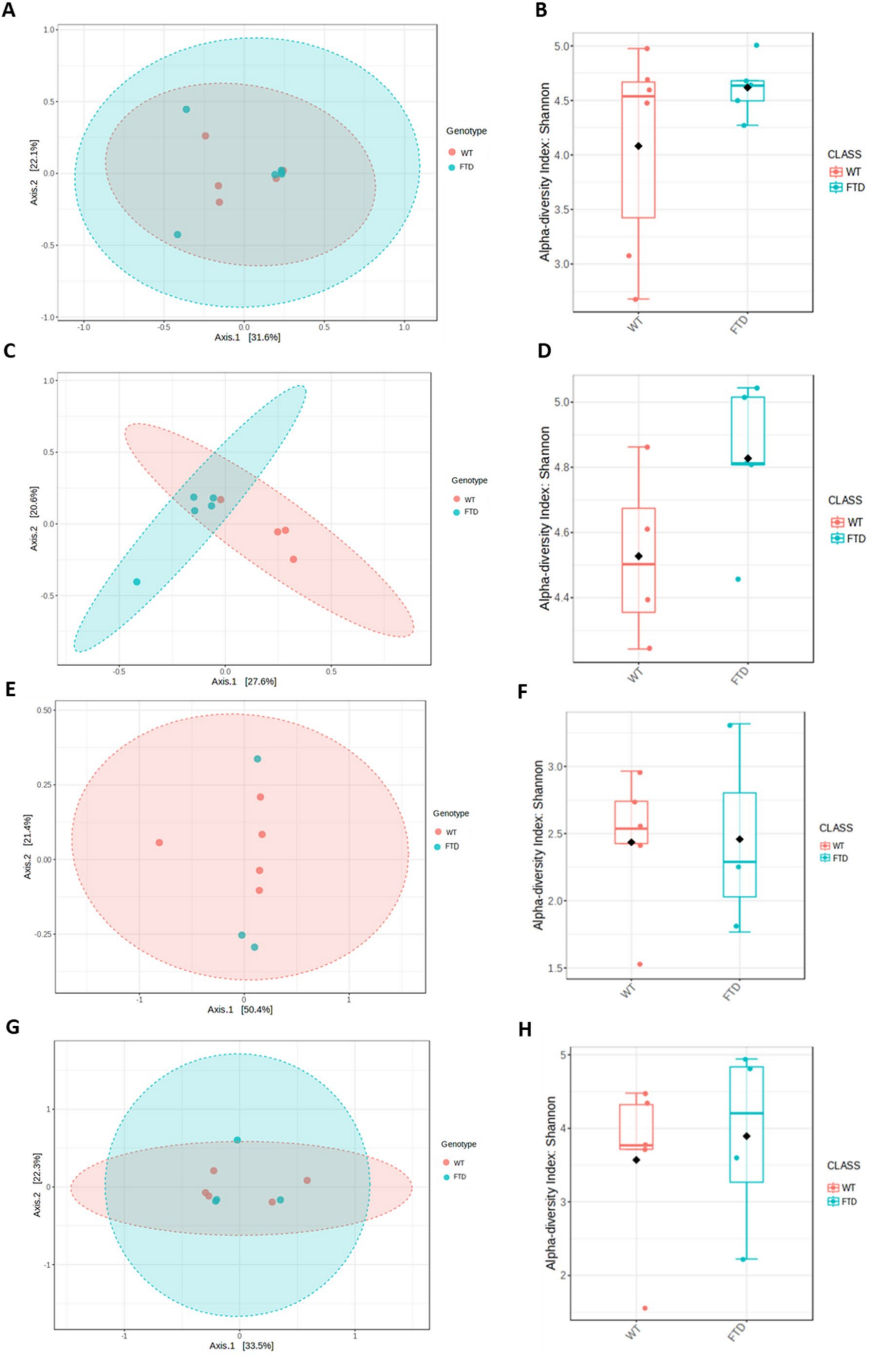
Microbial diversity. Principal coordinate analysis (PCoA) plots showing the distribution of microbial communities according to genotype in the feces (A), caecum (C), ileum (E), and jejunum (G). The axes indicate the percentage of variation explained by the first two principal coordinates. Shannon alpha‐diversity index is presented as boxplots to compare microbial diversity among genotypes in the feces (B), caecum (D), ileum (F), and jejunum (H).

We inspected the feces and the intestinal contents of the caecum, ileum and jejunum. Despite our study being underpowered for this kind of analyses, with *n* = 6 and 5 for WT and FTD mice, respectively, we noticed some potential differences to be confirmed in future studies. The PCoA analysis evidenced no difference in the beta‐diversity of WT and FTD mice in the feces (*p* = 0.898), ileum (*p* = 0.695) and jejunum (*p* = 0.832). However, in the caecum the two groups exhibited different beta‐diversity, as shown by the limited overlap of the two ellipses (*p* = 0.022) (Figure 5A,C,E,G). Shannon alpha‐diversity indexes showed no difference between the WT and FTD groups in feces (*p* = 0.24), caecum (*p* = 0.09), ileum (*p* = 0.95), and jejunum (*p* = 0.71) (Figure 5B,D,F,H).

**FIGURE 6:**
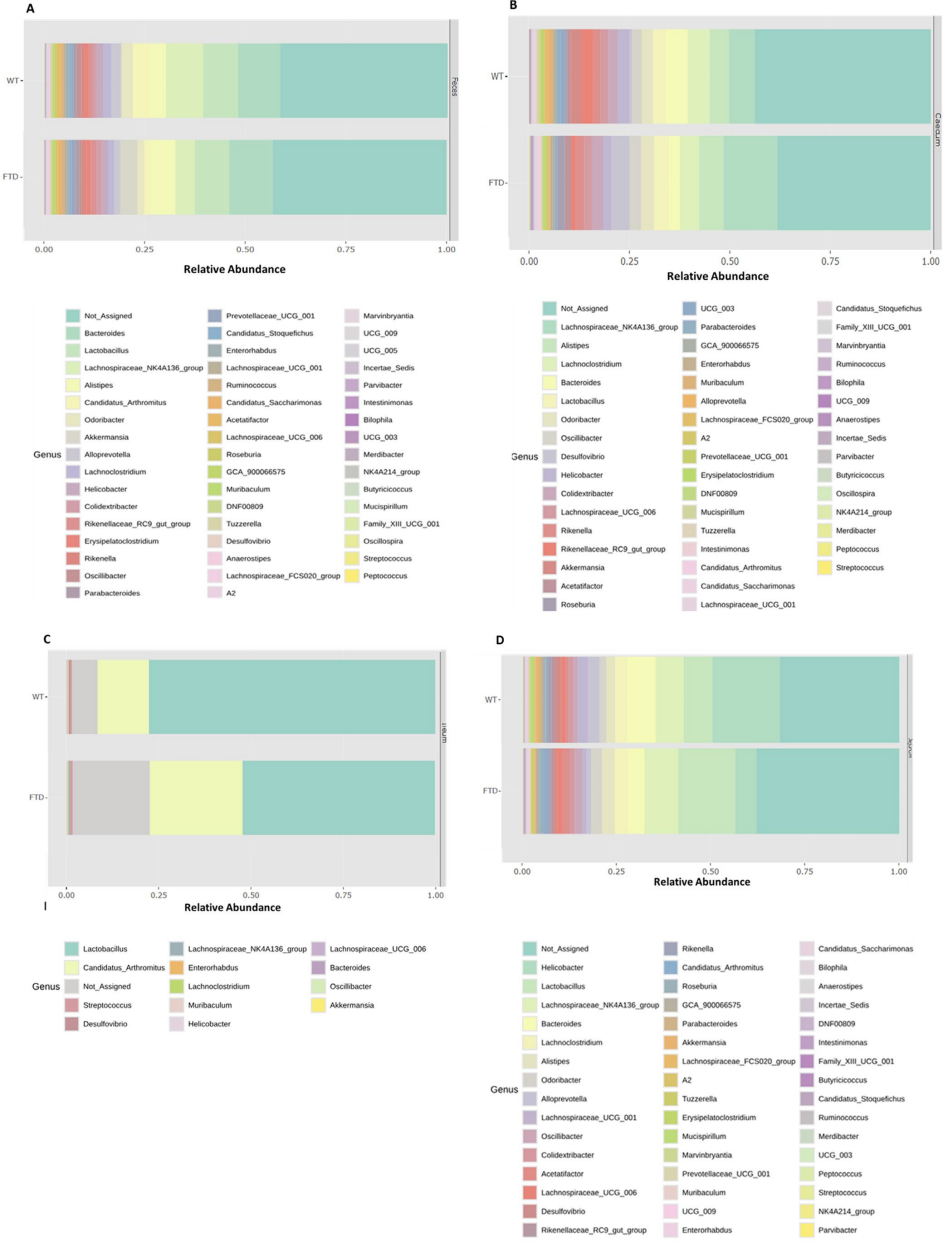
Relative abundance at the genus level. Stacked bar plots showing the relative abundance of bacterial genera in feces (A), caecum (B), ileum (C), and jejunum (D).

**FIGURE 7:**
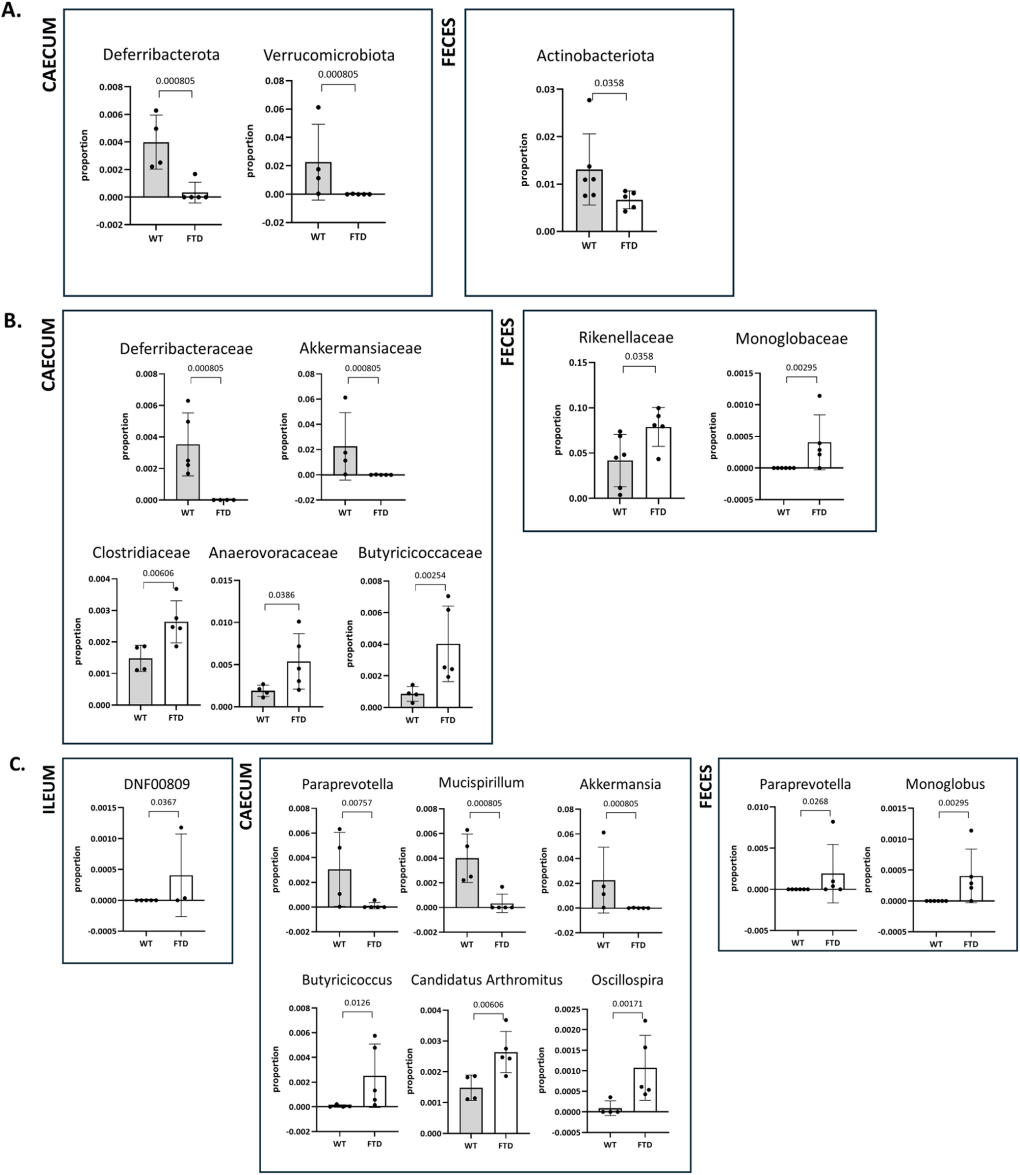
Phylum (A), family (B), and genus (C) changes in the intestinal contents and feces of FTD mice compared to WT mice (*p* values). Bar graphs illustrating the abundance of individual genera. Summary table available as Data S1.

We observed potential differences in phyla, families, and genera relative abundances between the two genotypes (Figure 6). These differences are more specifically shown in Figure 7.

Possible alterations in specific taxa within individual regions are shown in Figure 7 and Table S2. In the jejunum, there was no difference in relative abundance between the two genotypes at the phylum, family, and genus levels. In the ileum, there was no difference in relative abundance between the two genotypes at the phylum and family levels, while at the genus level we observed an increase in the genus *DNF00809* in FTD compared to WT mice (Figure 7C). In the caecum, we observed changes at the phylum level with the reduction in the relative abundance of *Verrucomicrobiota* and *Deferribacterota* in FTD mice (Figure 7A). At the family level, we observed a reduction in the relative abundance of *Deferribacteraceae* and *Akkermansiaceaa*, and an increase of that of *Clostridiaceae*, *Butyricicoccaceae* and *Anaerovoracaceae* in FTD mice (Figure 7B). At the genus level, we observed the reduction in the relative abundance of *Paraprevotella*, *Mucispirillum*, and *Akkermansia* and the increase in that of *Butyricicoccus*, *Candidatus_Arthromitus*, and *Oscillospira* in FTD mice (Figure 7C). In the feces, we observed the reduction of *Actinobacteria* at the phylum level in FTD mice (Figure 7A). At the family level, there was an increase in the abundance of *Rikenellaceae* and *Monoglobaceae* in FTD mice (Figure 7B). At the genus level, there was an increase in the abundance of *Paraprevotella* and *Monoglobus* in FTD mice compared to WT (Figure 7C).

### 3.4 FTD Mice Show Reduced Fecal SCFA Levels Compared to WT Mice

**FIGURE 8:**
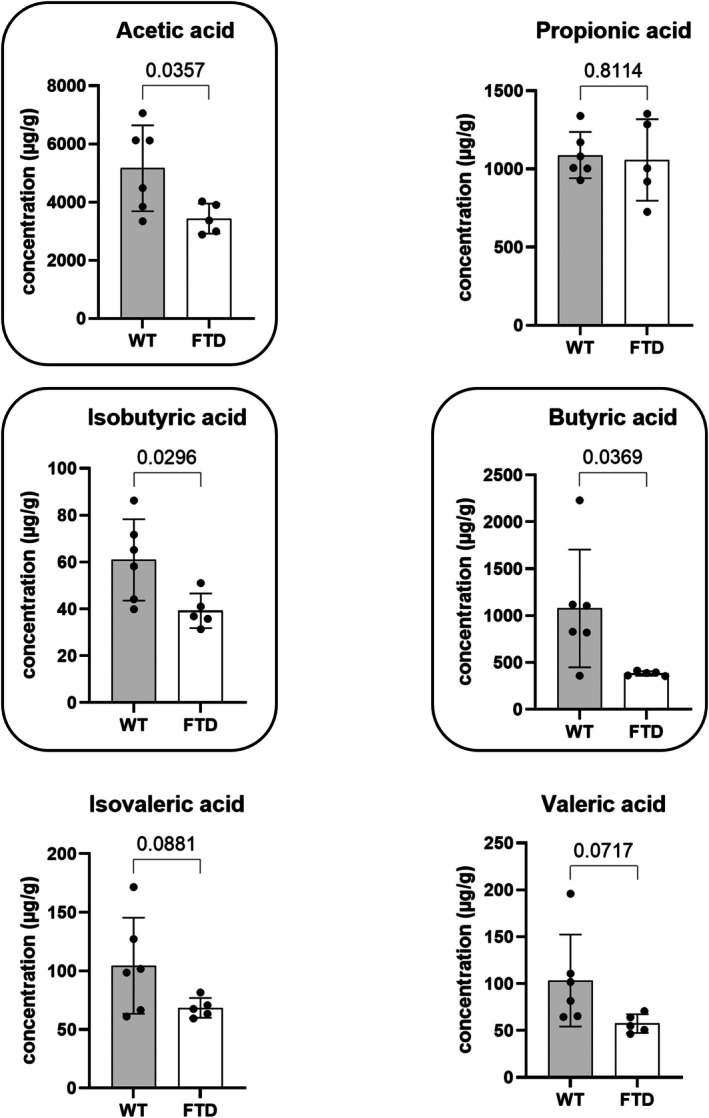
Fecal short‐chain fatty acid levels in FTD mice compared to WT mice. Unpaired *t*‐test, mean and SD are represented. WT group, *n* = 6, FTD group, *n* = 5.

Compared to WT mice, FTD mice showed a significant reduction in their fecal levels of acetic acid, isobutyric acid, and butyric acid, while isovaleric acid and valeric acid showed only a trend towards decrease. As for propionic acid, no difference was observed between the two groups (Figure 8).

## 4 Discussion

We previously investigated the brain eCB system in the TDP‐43 mouse model of FTD and, following the finding of decreased expression of the NAE‐degrading enzyme, FAAH, we treated these mice with a FAAH inhibitor, which improved many behavioral and histopathological alterations linked to FTD in this model [12], effects that we demonstrated in a follow‐up study to be mediated by the activation of CB1 and/or CB2 receptors [14]. In the current study, we focused on the small intestine of this TDP‐43 mouse model, since it has been reported that alterations of the gut‐brain axis, also through the loss of intestinal microbiome homeostasis (gut “dysbiosis”), are involved in several neurodegenerative and neuroinflammatory disorders [26].

First, we observed a reduction of the expression of *Gpr55* and *Dsg2* and an increase of *Pparg* expression in the duodenum. The two formers could contribute to dysregulating intestinal inflammation and integrity/permeability, and the latter may represent a negative feedback adaptation to these potential effects. Interestingly, gut microbiota have been shown to be causally associated with FTD [27], and we previously reported that GF mice exhibit reduced *Gpr55* expression in small intestine segments [19]. GPR55 in mice has been shown to be implicated in GI motility and inflammation and suggested to be involved in mucosal secretion and homeostasis [28]. On these bases, it is possible that the potential implication of intestinal GPR55 in FTD is modulated by the gut microbiota. Future experiments are needed to address the role of reduced GPR55 signaling in TDP‐43 mouse small intestine. As for *Dsg2*, its decrease is consistent with the “leaky gut” hypothesis in the context of age‐related diseases such as dementia [29].

*Gpr55* and *Pparg* expression changes were not sustained by any change in the known eCBome ligands of the receptors encoded by these genes and quantified here by HPLC‐MS/MS, that is, *N*‐palmitoyl‐ethanolamine (PEA) for GPR55, and AEA and DHEA for PPARγ. The duodenum of FTD mice also showed, however, reduced FA levels and increased FA‐derived oxylipins. Interestingly, the increased levels of all oxylipins were accompanied by the decrease of the levels of their respective precursors, that is, DGLA for 12‐HETrE and 15‐HETrE; AA for 8‐HETE, 11‐HETE, 12‐HETE, 12‐KETE, 15‐HETE, and 5,12‐DiHETE; EPA for 12‐HEPE and 15‐HEPE; and DHA for 14‐HDHA + 7‐HDHA and 17‐HDHA. This is consistent with the quantitative conversion of PUFAs into the corresponding oxylipins. Additionally, we also observed increased levels of both SPMs and pro‐inflammatory LTB4 in the duodenum, again suggestive of an altered inflammatory state in this section of the intestine in the TDP‐43 mice group. The reduced levels of LEA, which activate the anti‐inflammatory and intestinal permeability‐reducing PPARα, can also be interpreted in this sense.

No significant gene expression changes were observed in the jejunum, but as previously observed in the duodenum, the levels of some PUFAs and PUFA‐derived oxylipin were altered in TDP‐43 mice. In this case, changes in oxylipin levels were not necessarily accompanied by opposite changes in the corresponding PUFA precursors. Indeed, while 12‐HETE, 5‐HETE, 8‐HETE, 11‐HETE, 15‐HETE, and 5,12‐DiHETE were increased, AA was not significantly changed in the FTD group. The decrease of 9‐HODE was not associated with any changes in LA, and 12‐HETrE and 15‐HETrE increases were not associated with any change in DGLA. Similarly, the increases of 4‐HDHA, 14‐HDHA+7‐HDHA, 17‐HDHA, and 17‐oxo‐DHA were not associated with any change in DHA levels. This disparity between duodenum and jejunum may be explained by the differences in anatomy, function, pH, and reserves of precursor PUFAs between the two regions. On the other hand, 12‐KETE and 5,12‐DiHETE increases were associated with a significant increase of their direct biosynthetic precursor, 12‐HETE.

In the ileum, the increased expression of *Cnr1* in TDP‐43 mice was associated with increased levels of its ligands, such as AEA and (with much lower affinity) DHEA. The increased expression of *Gpr119* instead was accompanied by a reduction of its ligands, 2‐LG and 2‐OG. This is interesting because Gpr119 is known to play a role in the secretion of incretin hormones and incretins are currently under investigation to be potentially used as a new target in the treatment of neurodegenerative diseases [30]. The increased expression of *Napepld* was consistent with the trend towards increased levels of AEA, which could play a protective role in the ileum as it seems to do in the brain [12]. This adaptive response did not reach statistical significance as it may have been possibly mitigated by the concomitant increased expression of the AEA‐degrading enzyme, FAAH. It was recently shown that stabilizing NAPE‐PLD at the thiazide‐binding site is beneficial for curing hypertension [31]. Interestingly, hypertension is a risk factor for cognitive decline [32]. Thus, we can speculate that the potentially beneficial implication of intestinal NAPE‐PLD in FTD may be via its implication in hypertension. Finally, while most of the oxylipin changes observed in the ileum of the TDP‐43 group were not associated with changes in their PUFA precursor levels, the reduction of 13‐HODE‐G was accompanied by the significant reduction of its precursor, 2‐LG, in FTD mice.

In all the regions analyzed, oxylipin levels were altered in TDP‐43 mice, reflecting the oxidative state of the regions. This finding highlights the potential importance of PUFA‐derived oxidation products, whether enzymatically or nonenzymatically synthesized, in the pathophysiology of neurodegenerative disorders such as FTD. Both cerebral and peripheral oxidative stress have been associated with several brain conditions, such as stroke [33] and dementia [34], but little is known about oxylipin‐controlled free radical implication in FTD. However, oxylipins are known to be implicated in the modulation of neuroinflammation and to play a role in some neurodegenerative disorders, including dementias [35, 36]. Furthermore, it has been shown that several lipid metabolism abnormalities play a role in the pathophysiology of neurodegenerative diseases [37, 38].

Although underpowered due to the relatively low number of replicates, the analysis of the DNA of the intestinal content and feces suggested overall more microbiota changes in TDP‐43 mice in the lower part of the gastrointestinal tract. These preliminary results agree with the fact that microbial density within this tissue is lower in the proximal parts, and higher in its distal parts. All the alterations (except one in the ileum at the genus level) were observed in the caecum and feces. Gut microbiota, as well as some of their metabolites, have been demonstrated to be involved in several metabolic, psycho‐metabolic and mental disorders [17, 39, 40, 41]. As an important part of such metabolites, SCFAs have been extensively studied, and some of them are more characterized than others. These volatile lipophilic metabolites are known to be implicated in neurotransmission, neuroinflammation, neurometabolism and blood–brain barrier integrity [42]. Concerning their present quantification in the feces of FTD mice, they showed a statistically significant reduction of their levels in the case of butyric, isobutyric and acetic acid. Interestingly, these three SCFAs have been associated with good cognitive performance in mice [43] and humans [44], which suggests that the reduction of their levels in the TDP‐43‐FTD mouse model might be one of the causes of the lower cognitive performances of those mice, which is a feature also of FTD patients. Decreased SCFA levels have also been reported in other neurodegenerative disorders, such as Parkinson's disease [45] and Alzheimer's disease (AD) [46], and more generally they have also been associated with other mental disorders such as autism spectrum disorder (ASD), major depression, addiction‐related behaviors and schizophrenia [47]. As fecal SCFA levels represent only a portion of the total circulating SCFAs [47], their reduction in the feces of TDP‐43 mice might either be due to a reduction of their production by intestinal bacteria (due to a change in the diversity and composition of the gut microbiota) or to reduced excretion in the feces, with a bigger portion kept into the system. With this in mind, we thought it important to be careful with the interpretations of these data.

Butyric acid is one of the most studied SCFAs. It is known to have several effects on the host, such as contributing to the maintenance of colon health, improving glucose homeostasis, maintaining gut integrity by regulating tight junction proteins, and reducing inflammation [48]. It may also have additional regulatory effects on the production of neurotrophic factors, such as BDNF [47], whose activity is known to be impaired in the case of TDP‐43 abnormal function [49]. Here, reduced butyric acid levels in TDP‐43 mice, however, were not correlated to decreased abundance of known butyrate‐producing bacteria (data not shown), such as *Bacillus subtilis* [50] and *Eubacterium xylanophilum* [51]. Nevertheless, butyric acid has been described to be negatively correlated with anxiety and depression, which may be relevant to some of the psychiatric symptoms observed in FTD patients as well as in the TDP‐43‐FTD mouse model. It is even hypothesized that depression might be a predictor of early‐onset FTD neurodegeneration and hence represent the first symptom of dementia [52]. Depression is the most prevalent noncognitive comorbidity that occurs along with cognitive deficits and is associated with neurodegenerative disorders and cognitive decline [53]. Even though the association between depression and FTD is not well established, it was documented that depressive symptoms are the most frequent neuropsychiatric comorbidity of AD, another neurodegenerative disease, for which 50% of the patients are concerned [54]. Anxiety, which is the most frequent comorbidity associated with depression, is also frequently reported in the case of FTD patients [55]. Interestingly, Kalkan et al. [18] found that butyric acid reduces eCBome signaling, which reinforces the relevance of studying the eCBome and SCFAs together in the context of FTD.

Several studies showed that isobutyric acid, like butyric and acetic acid, is dysregulated in brain disorders. Indeed, in a valproic acid‐induced ASD rat model, the levels of these SCFAs were reduced in the feces of ASD rats compared to control rats nonexposed to valproic acid. These changes were also accompanied by changes in gut microbiota composition and neurotransmitter levels in the brain [56]. Another rodent study showed that mice receiving an FMT with feces from exercised mice would manifest an increase in their learning and memory skills associated with an increase in their caecal levels of SCFAs, including isobutyric acid, butyric acid, and acetic acid [57]. Another study, this time in human plasma, associated the reduction of these SCFAs with a diabetic cognitive impairment when compared to type‐2‐diabetic patients without cognitive symptoms [44].

Acetic acid, like butyric acid, is known to regulate food intake by acting on the hypothalamus [17]. This is interesting because it has been described that FTD patients have a dysregulated food intake. Indeed, eating abnormalities are present in up to 60% of patients with FTD and are one of the six core symptoms required for the diagnosis of behavioral variant FTD (bvFTD). The changes may include the following parameters: swallowing, appetite, stereotypic eating behavior and table manners, food preference (including sweet preference), and other oral behaviors such as food cramming and increased smoking [58]. Furthermore, a case–control study highlighted that early‐onset dementia (EOD) can be influenced by environmental factors and lifestyles, including diet. Indeed, in this study it was shown that adherence to a specific dietary pattern (MIND, Mediterranean‐DASH Intervention for Neurodegenerative Delay) may decrease the risk of EOD [59]. Recently, a reduction in acetate levels in mice has been shown to be associated with obesity and elevated levels of the eCB, 2‐AG, in the hypothalamus [17], again highlighting the potential importance of the gut microbiome‐eCBome axis, also in the context of metabolic and eating disorders with a neuroinflammatory component [40].

As it is well known, the diet influences gut microbiota composition and diversity [60]. Therefore, it makes sense that FTD patients with disturbed eating habits end up with gut dysbiosis. However, in the context of our FTD model, TDP‐43 mice were all fed with the same diet and did not display eating alterations (data not shown). Nevertheless, their gut microbiota was altered compared to WT mice. Indeed, in 2020 [61] it was shown that the gut microbiota composition of a genetically modified mouse model of amyotrophic lateral sclerosis (ALS) and FTD (*C9ORF72* mutation) plays an important role in the premature death of these mice and in the pathophysiology of FTD, by influencing the inflammatory response. When gut microbiota of FTD mice were targeted with broad spectrum antibiotics or fecal transplant, the inflammatory phenotypes of the mutant mice were attenuated. More generally and more recently, many studies demonstrated the implication of the gut microbiota in dementia disorders including FTD [27, 62]. Concerning the gut microbiota of our TDP‐43 mice, we preliminarily observed, in the feces, that *Paraprevotella* and *Monoglobus* were increased. Interestingly, it was previously shown that *Paraprevotella* was increased in mice with cognitive deficit in a model of AD [63]. Thus, species belonging to this genus may play a role in the pathophysiology of FTD. In contrast, a human study showed that *Monoglobus* abundance decreased in AD patients [64]. This may indicate either a species effect, or that this *genus* does not play a general key role in the pathophysiology of dementia.

Most of the microbiota taxonomic differences between TDP‐43 and WT mice that were suggested by our preliminary DNA sequencing results were observed in the caecum, in agreement with this region being the gastrointestinal section with the most bacterial diversity and abundance. In the caecum, we observed an increase in the relative abundance of the genera *Butyricicoccus, Oscillospira*, and *Candidatus Arthromitus*. Interestingly, *Butyricicoccus* was shown to be increased in an AD model of rats [65], which is consistent with our finding. In addition, *Oscillospira* was positively correlated with aging‐related symptoms [66], which is also in agreement with our findings, as FTD, like other dementias, is an age‐related disorder (early‐onset forms do not represent the majority of the cases). A study from 2019 showed that *Candidatus Arthromitus* is a specific inducer of differentiation in T helper 17 cells, which secrete the inflammatory cytokine IL‐17 [67] that was observed to be increased in FTD patients [68], results that are consistent with our findings. In the caecum we also showed a reduction in the abundances of *Paraprevotella* (contrary to what was observed in the feces), *Mucispirillum*, and *Akkermansia*. Consistent with our findings, *Mucispirillum* is reduced in mice with disrupted cognition [69] and has been reported to be negatively correlated with cognitive abilities and positively correlated with IL‐6 (pro‐inflammatory cytokine) levels in the brain cortex of the SAMP8 mouse model of dementia [70]. Indeed, a study on human circulating biomarkers of FTD published in 2015 reported an increase in the levels of plasmatic IL‐6 in 230 patients with FTD, independently of the clinical and genetic subtypes [71]. Moreover, in AD‐prone mice, *Mucispirillum* was strongly associated with immune inflammation [72]. In contrast, other studies showed a negative correlation between *Mucispirillum* and cognitive functions in AD mice [73], as well as with an increase in Aβ1–42‐induced AD mice [74]. *Mucispirillum* was also highlighted as a potential biomarker for insecticide exposure‐induced depression [75]. On the other hand, *Akkermansia* was increased in an AD mouse model with improved cognition [76], suggesting that cognitive impairment is probably associated with a reduction of *Akkermansia*, in agreement with our present finding of reduced *Akkermansia* relative abundance in TDP‐43 compared to WT mice. In the ileum, only the genus *DNF00809* showed a difference between the two genotypes, being increased in TDP‐43 mice. Unfortunately, this *genus* is not so well documented concerning its link with the brain and cognition. Only one study showed a link with aging (which could be extrapolated to reduced cognition), showing that this *genus* was abundant in young female rats compared to middle‐aged ones [77].

The above considerations on the chemotaxonomic differences in the gut bacteriomes of WT and TDP‐43 mice are somehow toned down by two important limitations of this study. First, and as mentioned above, we could only use a relatively low number of mice for each genotype (6 WT and 5 TDP‐43 mice), which certainly limited the statistical power of our analysis, although it did not prevent us from observing potential differences among intestinal sections from the same genotype, nor from finding some interesting differences in SCFA levels. Secondly, WT and TDP‐43 mice were not bred together, that is, they were not littermates, and, additionally, mice belonging to each genotype were not single‐housed. This may have generated artifactual differences between genotypes, such as those related to cage effects. However, despite these limitations, we believe that the observed differences reflect, at least to some significant extent, genotypic differences, in as much as they may explain some genotypic behavioral differences and are often in agreement with previous findings in other models of dementia and accompanying co‐morbidities. Further, they are sided by strong differences in eCBome signaling that, on the one hand, are instead unlikely to be due to cage effects, and, on the other hand, have been previously shown to often determine, or be determined by, gut microbiome alterations [19, 20, 40, 78].

## 5 Conclusions

Through the present study, we confirm the potential importance of the eCBome and of PUFA‐derived lipid mediators in the pathophysiology of FTD in the TDP‐43 mouse model of FTD. We also have reported potential genotype‐related alterations in gut microbiota and SCFAs in this model, which however need to be confirmed in future studies with a higher number of replicates. These findings highlight the complexity of the biological mechanisms involved in FTD, involving the gut bacteriome, SCFAs, the eCBome, oxylipins, and the gut‐brain axis. This work lays the foundations for future studies using this mouse model of FTD in order to investigate the microbiota‐gut‐lipid mediator‐brain axis as a target for the treatment of this disorder.

## Author Contributions

Vincenzo Di Marzo and Javier Fernandez‐Ruiz conceived and designed the research; Hayatte‐Dounia Mir, Giada Giorgini, Elizabeth Dumais, Irene Santos‐García, Carmen Rodríguez‐Cueto, and Eva de Lago performed the research and acquired the data; Cristoforo Silvestri supervised the project; Cristoforo Silvestri and Nicolas Flamand contributed to data curation; Hayatte‐Dounia Mir and Giada Giorgini analyzed the data. Hayatte‐Dounia Mir, Javier Fernandez‐Ruiz and Vincenzo Di Marzo interpreted the data. Hayatte‐Dounia Mir and Vincenzo Di Marzo wrote the original draft. All authors were involved in revising the manuscript.

## Funding

This work was financially supported by the Canada Research Excellence Chair on the Microbiome‐Endocannabinoidome Axis in Metabolic Health (CERC‐MEND) (to V.D.M., 760 2017‐2024), the Joint International Research Unit for Chemical and Biomolecular Studies on the Microbiome and its impact of Metabolic health and Nutrition (JIRU‐MicroMeNu), which in turn is funded by the Apogée program of the Canadian Federal Tri‐Agency through the Sentinel Nord programme of Université Laval, and two grants from the Spanish Ministry of Science and Innovation (RTI‐2018‐098885‐B‐100 and PID2021‐128906OB‐I00) with FEDER funds.

## Ethics Statement

All experiments were carried out in laboratory animals and were conducted according to local and European rules (directive 2010/63/EU) following the ARRIVE guidelines. All animal experiments were approved by the ethical committees of our university and the regulatory institution (PROEX 056/19 and PROEX 201.8/22).

## Conflicts of Interest

The authors declare no conflicts of interest.

## Supporting information


**Data S1:** fsb271306‐sup‐0001‐DataS1.docx.

## Data Availability

All data supporting the findings presented in this study are available within the article or accompanying materials. Raw data are available from the corresponding author upon reasonable request. Raw FASTQ files for 16S rDNA sequencing data have been deposited in the NCBI Sequence Read Archive (https://www.ncbi.nlm.nih.gov/bioproject/PRJNA1363213) under BioProject PRJNA1363213.
