# Supplementary material for: Defining the Intestinal eCBome and Oxylipin Signaling Systems in a TDP‐43 Mouse Model of Frontotemporal Dementia
Source: FASEB J. 2026 Jan 12;40(2):e71306. doi: 10.1096/fj.202502312RR (PMC12794397; doi:10.1096/fj.202502312RR)

**Supplementary Table 1. List of compounds quantified by HPLC-MS/MS and their change in FTD mice compared to WT mice.**

| Lipid Class | **Short Name** | **Complete Name** | **Duodenum** | **Jejunum** | **Ileum** |
| --- | --- | --- | --- | --- | --- |
| Prostaglandin | 6-keto PGF_1a_ | 6-keto Prostaglandin F_1α_ | ns | ↗ | ns |
|  | PGF_3a_ | Prostaglandin F_3α_ | NA | NA | NA |
|  | TXB_2_ | Thromboxane B_2_ | ns | ns | ns |
|  | PGE_3_ | Prostaglandin E_3_ | ns | ns | ns |
|  | PGF_2a_ | Prostaglandin F_2α_ | ns | ↗ | ns |
|  | PGE_1_ | Prostaglandin E_1_ | ns | ns | ns |
|  | PGE_2_ | Prostaglandin E_2_ | ns | ↗ | ns |
|  | PGD_2_ | Prostaglandin D_2_ | ↗ | ns | ns |
|  | 1a, 1b-dihomo PF_2a_ | 1a, 1b-dihomo Prostaglandin F_2α_ | ns | ns | **↘*** |
|  | 12(S)-HHTrE | 12S-hydroxy-heptadecatrienoic acid | ns | ns | ns |
|  | PGB_2_ | Prostaglandin B_2_ | NA | NA | NA |
|  | PGF_2a_-EA | Prostaglandin F_2a_ Ethanolamide | NA | NA | NA |
| Prostaglandin-Glycerol | PGF_2a_-G | Prostaglandin F_2a_-1-glyceryl ester | NA | NA | NA |
|  | PGE_2_-G | Prostaglandin E_2_-1-glyceryl ester | NA | NA | NA |
|  | PGD_2_-G | Prostaglandin D_2_-1-glyceryl ester | NA | NA | NA |
| Prostamide | PGE_2_-EA | Prostaglandin E_2_ Ethanolamide | NA | NA | NA |
|  | PGD_2_-EA | Prostaglandin D_2_ Ethanolamide | NA | NA | NA |
| N-Acyl-Ethanolamine (NAE) | SDEA | *N*-Stearidonoyl-Ethanolamine | NA | NA | NA |
|  | EPEA | *N*-Eicosapentaenoyl-Ethanolamine | NA | NA | NA |
|  | LEA | *N*-Linoleoyl-Ethanolamine | **↘*** | ns | ns |
|  | DHEA | *N*-Docosahexaenoyl-Ethanolamine | ns | ns | ↗ |
|  | AEA | *N*-Arachidonoyl-Ethanolamine | ns | ns | ↗ |
|  | PEA | *N*-Palmitoyl-Ethanolamine | ns | ns | ns |
|  | OEA | *N*-Oleoyl-Ethanolamine | ns | ns | ns |
|  | SEA | *N*-Stearoyl-Ethanolamine | ns | **↗*** | ns |
|  | DPEA(n-6) | *N*-Docosapentaenoyl-Ethanolamine (omega-6) | ns | ns | ns |
|  | DPEA(n-3) | *N*-Docosapentaenoyl-Ethanolamine (omega-3) | ns | ns | ns |
| Monoacyl-glycerol (MAG) | 1/2-SDG | 1/2-Stearidonoyl-Glycerol | NA | NA | NA |
|  | 1/2-EPG | 1/2-Eicosapentaenoyl-Glycerol | ns | ns | ns |
|  | 1/2-DHG | 1/2-Docosahexaenoyl-Glycerol | ↘ | ns | ns |
|  | 1/2-LG | 1/2-Linoleoyl-Glycerol | ns | ns | **↘*** |
|  | 1/2-AG | 1/2-Arachidonoyl-Glycerol | ↘ | ns | ns |
|  | 1/2-DPG | 1/2-Docosapentaenoyl-Glycerol | ns | ns | ns |
|  | 1/2-OG | 1/2-Oleoyl-Glycerol | ns | ↗ | **↘*** |
|  | 1/2-PG | 1/2-Palmitoyl-Glycerol | ns | ns | ns |
| Fatty acid | SDA | Stearidonic Acid | ↘ | ns | ns |
|  | EPA | Eicosapentaenoic Acid | **↘*** | ns | ns |
|  | DHA | Docosahexaenoic Acid | **↘*** | ns | ns |
|  | AA | Arachidonic Acid | **↘*** | ↗ | ns |
|  | DPA (n-3) | Docosapentaenoic Acid (omega-3) | ns | ns | ns |
|  | DPA (n-6) | Docosapentaenoic Acid (omega-6) | ns | ns | ns |
|  | LA | Linoleic Acid | **↘*** | ns | ns |
|  | OA | Oleic Acid | ns | **↗*** | ns |
|  | DGLA | Dihomo-γ-Linolenic Acid | ↘ | ns | ns |
| NAE-derived Oxylipin | 15-HEPE-EA | 15-hydroxy-Eicosapentaenoic acid Ethanolamide | NA | NA | NA |
|  | 13-HODE-EA | 13-Hydroxy-Octadecadienoic acid Ethanolamide | ns | ns | **↘*** |
|  | 15-HETE-EA | 15-Hydroxy-Eicosatetraenoic acid Ethanolamide | NA | NA | NA |
|  | 17-HDPA-EA | 17-Hydroxy-Docosapentaenoic acid Ethanolamide | NA | NA | NA |
|  | 17-HDHA-EA | 17-Hydroxy-Docosahexaenoic acid Ethanolamide | NA | NA | NA |
|  | 5-HETE-EA | 5-Hydroxy-Eicosatetraenoic acid Ethanolamide | NA | NA | NA |
|  | 5-KETE-EA | 5-oxo-Eicosatetraenoic acid Ethanolamide | NA | NA | NA |
| MAG-derived Oxylipin | 15-HEPE-G | 15-Hydroxy-Eicosapentaenoic acid Glycerol | NA | NA | NA |
|  | 12-HEPE-G | 12-Hydroxy-Eicosapentaenoic acid Glycerol | NA | NA | NA |
|  | 13-HODE-G | 13-Hydroxy-Octadecadienoic acid Glycerol | ns | ns | **↘*** |
|  | 15-HETE-G | 15-Hydroxy-Eicosatetraenoic acid Glycerol | NA | NA | NA |
|  | 17-HDPA-G | 17-Hydroxy-Docosapentaenoic acid Glycerol | NA | NA | NA |
|  | 5-HETE-G | 5-Hydroxy-Eicosatetraenoic acid Glycerol | ns | **↗*** | ns |
|  | 5-KETE-G | 5-oxo-Eicosatetraenoic acid Glycerol | ns | ns | ns |
| Fatty acid-derived Oxylipin | 18-HEPE | 18-Hydroxy-Eicosapentaenoic acid | ns | ns | **↗*** |
|  | 8(S),15(S)-DiHETE | 8S,15S-Dihydroxy-Eicosatetraenoic acid | NA | NA | NA |
|  | 5(S),15(S)-DiHETE | 5S,15S-Dihydroxy-Eicosatetraenoic acid | NA | NA | NA |
|  | 13(S)-HOTrE | 13S-Hydroxy-Octadecatrienoic acid | ns | ↗ | ns |
|  | 15-HEPE | 15-Hydroxy-Eicosapentaenoic acid | **↗*** | NA | NA |
|  | 12-HEPE | 12-Hydroxy-Eicosapentaenoic acid | **↗*** | **↗*** | ns |
|  | 12-HETE | 12-Hydroxy-Eicosatetraenoic acid | **↗*** | **↗*** | ns |
|  | 9-HODE | 9-Hydroxy-Octadecadienoic acid | ns | **↘*** | **↘*** |
|  | 13-HODE | 13-Hydroxy-Octadecadienoic acid | ns | ns | **↘*** |
|  | 15-HETE | 15-Hydroxy-Eicosatetraenoic acid | **↗*** | **↗*** | ns |
|  | 13-KODE | 13-oxo-Octadecadienoic acid | ns | ns | ns |
|  | 15-KETE | 15-oxo-Eicosatetraenoic acid | ns | ns | ns |
|  | 11-HETE | 11-Hydroxy-Eicosatetraenoic acid | **↘*** | **↗*** | ns |
|  | 8-HETE | 8-Hydroxy-Eicosatetraenoic acid | **↘*** | **↗*** | ns |
|  | 17-HDHA | 17-Hydroxy-Docosahexaenoic acid | **↗*** | **↗*** | ns |
|  | 14-HDHA + 7-HDHA | 14- and 7-Hydroxy-Docosahexaenoic acid | **↗*** | **↗*** | ns |
|  | 14-HDHA + 7-HDHA | 14- and 7-Hydroxy-Docosahexaenoic acid | **↗*** | **↗*** | ns |
|  | 4-HDHA | 4-Hydroxy-Docosahexaenoic acid | ns | ns | ns |
|  | 12-KETE | 12-oxo-Eicosatetraenoic acid | **↗*** | **↗*** | ns |
|  | 5-HETE | 5-Hydroxy-Eicosatetraenoic acid | ns | **↗*** | ns |
|  | 17-HDPA | 17-Hydroxy-Docosapentaenoic acid | NA | NA | ns |
|  | 17-oxo-DHA | 17-oxo-Docosahexaenoic Acid | NA | **↗*** | ns |
|  | 15-HETrE | 15-Hydroxy-Eicosatrienoic acid | **↗*** | **↗*** | ns |
|  | 12-HETrE | 12-Hydroxy-Eicosatrienoic acid | **↗*** | **↗*** | ns |
|  | 5-KETE | 5-oxo-Eicosatetraenoic acid | ns | ns | ns |
|  | 15(S)-HpEDE | 15S-hydroperoxy-Eicosadienoic acid | ↘ | ns | ns |
|  | 5(S),12(S)-DiHETE | 5S,12S-Dihydroxy-Eicosatetraenoic acid | ns | **↗*** | NA |
| Specialized pro-resolving mediator (SPM) | RVE_1_ | Resolvin E_1_ | NA | NA | NA |
|  | RVD_3_ | Resolvin D_3_ | NA | NA | NA |
|  | RVD_2_ | Resolvin D_2_ | NA | NA | NA |
|  | RVD_1_ | Resolvin D_1_ | NA | NA | NA |
|  | RVD_4_ | Resolvin D_4_ | NA | NA | NA |
|  | RVE_4_ | Resolvin E_4_ | **↗*** | **↗*** | ns |
|  | Maresin 1 | 7R,14S-Dihydroxy-Docosahexaenoic acid | NA | NA | NA |
|  | 10,17-DiHDHA (PDX) | 10,17-Dihydroxy-Docosahexaenoic acid (Protectin DX) | **↗*** | ↗ | ns |
|  | RVD_5_ | Resolvin D_5_ | NA | ns | NA |
|  | Maresin 2 | 13R,14S-Dihydroxy-Docosahexaenoic acid | NA | NA | NA |
| Leukotriene | 5(S),6(R)-DiHETE | 5S,6R-Dihydroxy-Eicosatetraenoic acid | NA | NA | NA |
|  | 20-COOH-LTB_4_ | 20-carboxy Leukotriene B_4_ | NA | NA | NA |
|  | 20-OH-LTB_4_ | 20-hydroxy Leukotriene B_4_ | NA | NA | NA |
|  | EXC_4_ | Eoxin C_4_ (14,15-Leukotriene C_4_) | NA | NA | NA |
|  | EXD_4_ | Eoxin D_4_ (14,15-Leukotriene D_4_) | NA | NA | NA |
|  | LTC_4_ | Leukotriene C_4_ | NA | NA | NA |
|  | LTD_4_ | Leukotriene D_4_ | NA | NA | NA |
|  | LTE_4_ | Leukotriene E_4_ | NA | NA | NA |
|  | LTB_5_ | Leukotriene B_5_ | NA | NA | NA |
|  | LTB_4_ | Leukotriene B_4_ | **↗*** | **↗*** | ns |
|  | 12-oxo LTB_4_ | 12-oxo Leukotriene B_4_ | NA | NA | NA |
|  | LTB_3_ | Leukotriene B_3_ | NA | NA | NA |
|  | EXC_4_-EA | Eoxin C_4_ Ethanolamide | NA | NA | NA |
|  | EXC_4_-G | Eoxin C_4_ Glycerol | NA | NA | NA |

| NA | non applicable (n too low or non detected at all) |
| --- | --- |
| ns | non significant |
| ↗ | statistical trend increase in FTD mice |
| ↘ | statistical trend decrease in FTD mice |
| **↗*** | significant increase in FTD mice |
| **↘*** | significant decrease in FTD mice |

|  | **Phylum level** | **Direction of the change in FTD mice** |
| --- | --- | --- |
| Jejunum | No change | - |
| Ileum | No change | - |
| Caecum | Verrucomicrobiota (p=0.0008)  Deferribacterota (p=0.0008) | Decreased |
| Feces | Actinobacteria (p=0.036) | Decreased |
|  | **Family level** | **Direction of the change in FTD mice** |
| Jejunum | No change | - |
| Ileum | No change | - |
| Caecum | Deferribacteraceae (p= 0.0008)  Akkermansiaceaa (p= 0.0008) | Decreased |
|  | Clostridiaceae (p= 0.0061)  Butyricicoccaceae (p= 0.0025)  Anaerovoracaceae (p= 0.0386) | Increased |
| Feces | Rikenellaceae (p=0.036)  Monoglobaceae (p=0.003) | Increased |
|  | **Genus level** | **Direction of the change in FTD mice** |
| Jejunum | No change | - |
| Ileum | DNF00809 (p= 0.0367) | Increased |
| Caecum | Paraprevotella (p= 0.0076)  Mucispirillum (p= 0.0008)  Akkermansia (p= 0.0008) | Decreased |
|  | Oscillospira (p= 0.0017)  Candidatus_Arthromitus (p= 0.0061)  Butyricicoccus (p= 0.0126) | Increased |
| Feces | Paraprevotella (p=0.026)  Monoglobus (p=0.003) | Increased |

**Supplementary Table 2. Phylum, family and genus changes in FTD mice compared to WT mice in the intestines and feces (p values). Graphs available in the Supplementary figures.**

| **Gene name** | **Primer 1 sequence (5'-3')** | **Primer 2 sequence (5'-3')** |
| --- | --- | --- |
| *Gapdh* | AATGGTGAAGGTCGGTGTG | GTGGAGTCATACTGGAACATGTAG |
| *Cnr1* | GGGCACCTTCACGGTTCTG | GTGGAAGTCAACAAAGCTGTAGA |
| *Cnr2* | GCTCTTGGGACCTACGTG | GCTTTGGCTTCTTCTACTGGAG |
| *Ppara* | AGAGCCCCATCTGTCCTCTC | ACTGGTAGTCTGCAAAACCAAA |
| *Pparg* | GAATTAGATGACAGTGACTTGGC | AGCAGGTTGTCTTGGATGTC |
| *Trpv1* | GTGGACAGCTACAGTGAGATAC | GCCACATACTCCTTGCGAT |
| *Gpr18* | GTGGTGTTTTACCCAAGCCTC | TGGTCAGGGTCATTACCCAGA |
| *Gpr55* | CTGGCAGTCCATATCCCCAC | GCACCAGCAGTAAATCGAAAACA |
| *Gpr119* | CTTGCTGTCCTAACCATCCTCA | CCACGCCAATCAAGGTATCAG |
| *Faah* | ACTTGGACGTGGTGCTAACC | GCCTATACCCTTTTTCATGCCC |
| *Napepld* | CCATCCGAGCTTATGAACCAA | GCCACAGATCTCTTTGTTTGAAG |
| *Mgll* | TGTCCTGCCAAATATGACCTT | ATGATTCCATGAGCAGGTAGG |
| *Alox5* | CCATCTGCCTGCTATATAAGAACC | CCAGTCGTACTTTGAATCCGT |
| *Alox12* | GCTCCAATTCCATTTGCTGAAC | GCTTCGCGTGTTAATTTCCATAG |
| *Tjp1* | GAGCGGGCTACCTTACTGAAC | GTCATCTCTTTCCGAGGCATTAG |
| *Dsg2* | GGAAACGGACTTCACTTAGAGG | TGGCAATCGGGTTCTTTCTGG |
| *Ocln* | CTGGATCTATGTACGGCTCACA | TCCACGTAGAGACCAGTACCT |
| *Cdh1* | CTCCAGTCATAGGGAGCTGTC | TCTTCTGAGACCTGGGTACAC |
| *Tnfa* | GATGAGAAGTTCCCAAATGGC | ACTTGGTGGTTTGCTACGA |
| *Il1b* | GACCTGTTCTTTGAAGTTGACG | CTCTTGTTGATGTGCTGCTG |

**Supplementary Table 3. Sequences of the primers used for qPCRs.**


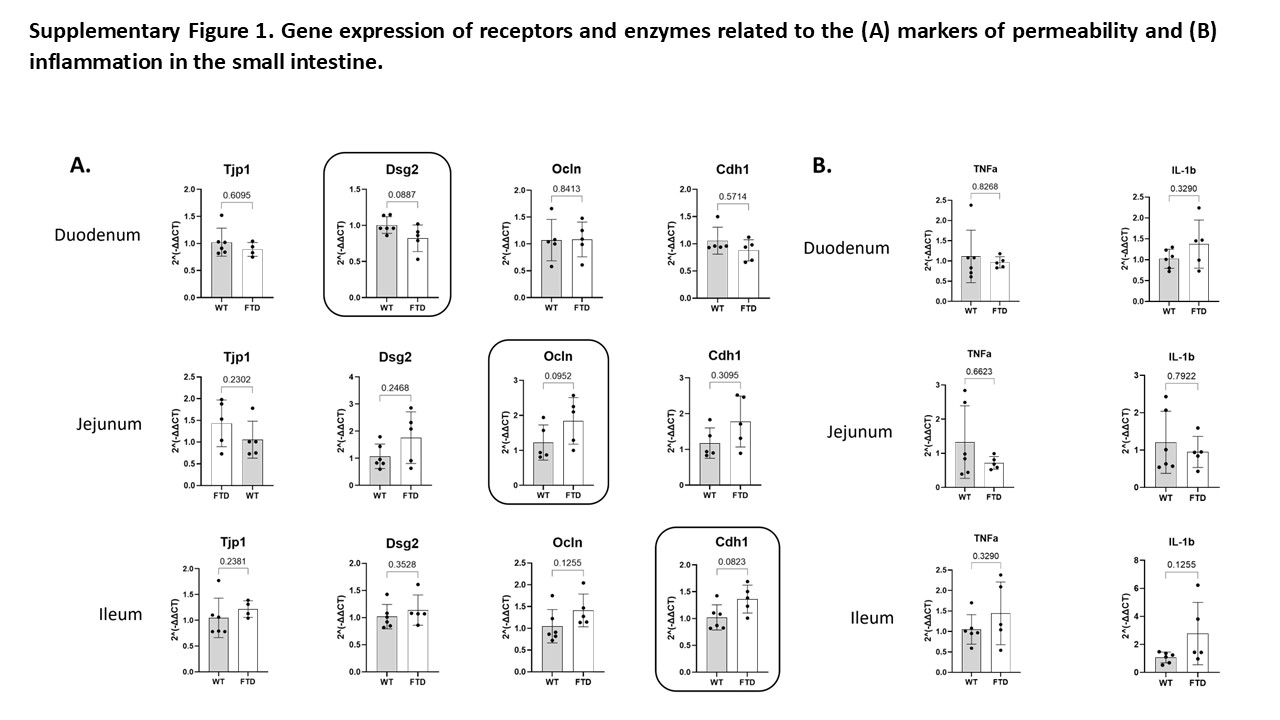

Supplement: Supplementary file 1 — Data S1: fsb271306‐sup‐0001‐DataS1.docx. [file FSB2-40-e71306-s001.docx]
